# Risk factors for fecal incontinence after surgery for cryptoglandular anal fistula: Protocol of a meta-analytic study

**DOI:** 10.1371/journal.pone.0351563

**Published:** 2026-06-09

**Authors:** Cheng Tang, Zubing Mei

**Affiliations:** 1 Department of Anorectal Surgery, Shanghai Baoshan Hospital of Integrated Traditional Chinese and Western Medicine, Shanghai, China; 2 Department of Anorectal Surgery, Shuguang Hospital, Shanghai University of Traditional Chinese Medicine, Anorectal Disease Institute of Shuguang Hospital, Shanghai, China; Sichuan University, CHINA

## Abstract

**Introduction:**

Fecal incontinence after anal fistula surgery is a feared functional complication that can impair dignity, social participation, and quality of life. Reported rates vary widely across procedures and fistula phenotypes, and individual studies suggest multiple patient-, disease-, and surgery-related predictors. However, the prognostic evidence remains dispersed, with inconsistent outcome definitions and variable adjustment for confounding. This protocol describes a systematic review and quantitative synthesis to identify factors associated with postoperative fecal incontinence after surgery for cryptoglandular anal fistula.

**Methods and analysis:**

The study will follow the Preferred Reporting Items for Systematic Review and Meta-Analysis Protocols guidance. PubMed, Embase, Cochrane Library, Web of Science, and grey-literature sources will be searched from inception to January 11, 2026, without language restrictions. Eligible studies will be cohort or case-control studies of adults undergoing surgery for cryptoglandular anal fistula that report new-onset or worsening postoperative fecal incontinence using validated scores or clearly defined clinical criteria at least 3 months after surgery. Two reviewers will independently screen records, extract data, and assess risk of bias using the Quality in Prognosis Studies tool and, where applicable, the Newcastle-Ottawa Scale. Adjusted estimates will be prioritized for random-effects meta-analysis when studies are clinically comparable. Heterogeneity and confounding will be explored by fistula complexity, procedure type, follow-up duration, and baseline continence assessment. Certainty of evidence will be rated using a prognostic-factor approach.

**Discussion:**

By integrating observational evidence across diverse surgical strategies, this review aims to generate pooled, evidence-based prognostic estimates for postoperative fecal incontinence. The findings may support preoperative counseling, baseline continence assessment, individualized procedure selection, sphincter imaging where appropriate, and targeted strategies to reduce preventable functional harm.

**Ethics and Dissemination:**

No ethical approval is required as this study uses existing data. Results will be disseminated through peer-reviewed journals and conferences to inform anal fistula management.

**Registration:**

International Prospective Register of Systematic Reviews (CRD420261283433)

## Introduction

Anal fistula is a chronic tract connecting the anal canal to perianal skin, most commonly arising from cryptoglandular infection [1]. Although historical population data estimated an incidence around 8–9 per 100,000 persons annually, the true burden is likely higher in contemporary practice given improved detection and referral patterns [2]. A large UK database study has provided more recent population-representative estimates, reporting point prevalence on the order of 1–2 per 10,000 patients in recent years [3].

Operative management remains the mainstay for cryptoglandular fistula, with the overarching goal of eradicating sepsis while preserving continence. International guidelines emphasize this balance, recommending lay-open approaches (e.g., fistulotomy) only when sphincter division is expected to be limited, and favoring sphincter-preserving options when the anticipated functional risk is substantial [4,5]. The continence trade-off is clinically consequential: even “minor” leakage can meaningfully affect daily living, intimacy, and mental health, and may persist long after the surgical wound has healed [6,7].

Postoperative fecal incontinence is particularly relevant in anal fistula surgery because the procedures inherently interact with the continence apparatus. Fistulotomy, for example, achieves excellent healing for appropriately selected low tracts but can compromise sphincter integrity if applied to higher or complex fistulas [8,9]. The American Society of Colon and Rectal Surgeons notes that fistulotomy for high-lying or complex fistulas can be followed by substantial rates of postoperative fecal incontinence, and highlights specific groups at heightened risk (including female patients, those with recurrent disease, complex anatomy, prior anorectal surgery, or baseline continence impairment) [4,5]. These concerns underpin the modern shift toward sphincter-preserving strategies, such as ligation of the intersphincteric fistula tract (LIFT) or advancement flap--in selected patients, where comparative evidence suggests similar healing in some contexts with potentially better continence preservation for less disruptive procedures [10,11].

Multiple primary studies have proposed predictors of postoperative fecal incontinence after fistula operations. Classic observational work identified anatomic and tract-related contributors including higher internal openings, posterior openings, and secondary extensions were associated with subsequent fecal incontinence after fistulotomy [12–15]. Larger series examining mixed procedures have also linked impaired continence to fistula height, female sex, previous fistula surgery, and operative approach--findings that remain clinically intuitive given cumulative sphincter injury and scarring with repeat interventions [16]. In a well-cited cohort from a tertiary setting, older age and high transsphincteric or suprasphincteric tracts were independent predictors of new postoperative fecal incontinence, supporting the notion that baseline muscle reserve and the extent of sphincter involvement both matter [17].

Beyond categorical “procedure type,” there is increasing interest in quantifying sphincter injury. Endoanal ultrasonography work suggests that increasing length of sphincter division correlates with postoperative symptoms, reinforcing the need for preoperative mapping and conservative selection for lay-open approaches [18]. This concept is also consistent with guideline framing: the more sphincter divided, the more the balance tilts from cure toward functional compromise, particularly in patients with occult obstetric injury or prior anorectal procedures [4,5].

A major challenge in the postoperative fecal incontinence literature is heterogeneity in outcome definition and ascertainment. Fecal incontinence may be reported as a binary event, graded by clinician judgement, or quantified using validated severity instruments. Widely used measures include the Cleveland Clinic Florida (Wexner) score and the Vaizey or St Mark’s score, which provide reproducible symptom-based grading and facilitate comparison across studies, though they differ in domains such as urgency and medication use [19,20]. Inconsistent follow-up timing also complicates interpretation: early seepage related to wound healing may differ from persistent fecal incontinence that reflects durable sphincter dysfunction. Additionally, observational studies variably adjust for baseline continence, fistula complexity, and prior pelvic floor injury, making it difficult to distinguish true prognostic factors from confounded associations.

Despite the clinical importance, prognostic evidence on postoperative fecal incontinence after cryptoglandular fistula surgery has not been consolidated in a way that prioritizes risk prediction. Existing syntheses in anal fistula largely concentrate on technique efficacy (healing or recurrence) or compare specific operations, rather than systematically evaluating prognostic factors across patient, disease, and treatment domains [21–24]. As a result, perioperative counseling often relies on general risk statements (e.g., “higher fistula equals higher risk”) without robust, pooled effect estimates that could support individualized decision-making, such as whether a borderline tract should undergo staged management, a sphincter-sparing approach, or modified lay-open with adjunctive repair. This review will explicitly position itself as a prognostic factor synthesis, with fecal incontinence as the primary outcome, and will differentiate itself from prior meta-analyses that emphasize healing, recurrence, and procedural comparisons.

Therefore, this study will systematically identify and quantitatively synthesize observational evidence on factors associated with postoperative fecal incontinence after surgery for cryptoglandular anal fistula. In addition to pooling effect estimates where appropriate, we will appraise the credibility and certainty of evidence for each candidate factor using established methods for prognostic-factor reviews [25]. The resulting evidence map is intended to aid risk stratification, inform shared decision-making, and highlight research gaps, particularly the need for standardized fecal incontinence definitions, baseline continence measurement, transparent reporting of fistula complexity, and explicit reporting of sphincter involvement and division.

## Methods

### Registration and reporting standards

This protocol has been prepared in accordance with the Preferred Reporting Items for Systematic Review and Meta‑Analysis Protocols (PRISMA‑P) statement [26]. The conduct of the review will follow the methodological guidance provided in the Cochrane Handbook for Systematic Reviews of Interventions [27], and the final report will adhere to the PRISMA 2020 checklist. The protocol was prospectively registered on PROSPERO (registration number CRD420261283433) prior to the commencement of formal study screening and data extraction. Any deviations from the registered protocol will be documented with the date of change, a clear rationale, and an assessment of their potential influence on the review’s findings.

### Eligibility criteria

Eligibility criteria were defined a priori based on a modified PICOS (Population, Index prognostic factors, Comparator, Outcomes, Study design) framework.

### Population

The study population will consist of adults (≥18 years) who have undergone surgical treatment for cryptoglandular anal fistula (fistula‑in‑ano). Studies focusing exclusively on the following populations will be excluded: fistulas associated with Crohn’s disease, cohorts defined by obstetric sphincter injury without concomitant fistula surgery, pediatric patients, and those managed non‑surgically. Studies with mixed etiologies will be eligible only if data pertaining to cryptoglandular fistulas can be extracted separately or if this subgroup constitutes the vast majority (>90%) of the sample.

### Index prognostic factors

We will examine patient‑, fistula‑, and procedure‑related factors reported as potential predictors in the primary literature (**Table 1**). Prespecified factors of interest include, but are not limited to:

**Table 1:** Candidate prognostic factors for postoperative fecal incontinence after anal fistula surgery.

Risk factor	Category	Definition/description	Typical measurement method	Source/reference
Female sex	Patient-related	Higher susceptibility to postoperative fecal incontinence may reflect obstetric sphincter injury, pelvic floor differences, and lower baseline reserve	Female vs male (binary)	Garcia-Aguilar et al. reported incontinence associated with female sex. (Garcia-Aguilar J, Belmonte C, Wong WD, et al. Anal fistula surgery. Factors associated with recurrence and incontinence. Dis Colon Rectum. 1996;39(7):723−9.)
Older age	Patient-related	Age-related decline in sphincter strength/neuromuscular function may reduce compensatory capacity after surgery	Years; commonly analyzed as continuous or categorical (e.g., > 45 years)	Older age associated with higher postoperative incontinence in prognostic analyses. (Abbas MA, Jackson CH, Haigh PI. Predictors of outcome for anal fistula surgery. Arch Surg. 2011;146(9):1011−6.)
Baseline continence status	Patient-related	Pre-existing fecal incontinence or reduced control increases likelihood of “worsening” after sphincter-involving procedures	Wexner/CCF-IS or Vaizey/St Mark’s at baseline; baseline fecal incontinence present vs absent	Standard scoring systems widely used for baseline assessment and follow-up comparison. (Jorge JM, Wexner SD. Etiology and management of fecal incontinence. Dis Colon Rectum. 1993;36(1):77–97.)
Prior fistula surgery/redo setting	Patient-related	Repeat interventions may cause cumulative sphincter trauma and scarring	History of fistula operation (yes/no); number of prior procedures	Prior fistula surgery associated with postoperative incontinence in large observational series. (Garcia-Aguilar J, Belmonte C, Wong WD, et al. Anal fistula surgery. Factors associated with recurrence and incontinence. Dis Colon Rectum. 1996;39(7):723−9.)
High/complex fistula anatomy	Fistula-related	Greater sphincter involvement (e.g., high transsphincteric/suprasphincteric) increases functional risk	Parks classification; “high” vs “low”; MRI/EAUS/TRUS mapping	High (trans-/supra-sphincteric) fistulas strongly predicted incontinence vs low/subcutaneous in adjusted models. (Abbas MA, Jackson CH, Haigh PI. Predictors of outcome for anal fistula surgery. Arch Surg. 2011;146(9):1011−6.)
Posterior internal opening/secondary extensions	Fistula-related	Certain locations and extensions may require broader division or are markers of more complex disease	Opening location (anterior/posterior); presence of extensions on imaging/operative findings	High opening, posterior opening, and extensions linked to postoperative fecal incontinence after fistulotomy. (van Tets WF, Kuijpers HC. Continence disorders after anal fistulotomy. Dis Colon Rectum. 1994;37(12):1194−7.)
Horseshoe configuration/multiple tracts	Fistula-related	Horseshoe or branching patterns often indicate extensive disease and higher operative burden	Horseshoe (yes/no); multiple tracts on MRI/EAUS; operative description	Horseshoe extension and complex type were associated with adverse outcomes; postoperative fecal incontinence also tracked with “high”/complex disease and technique. (Garcia-Aguilar J, Belmonte C, Wong WD, et al. Anal fistula surgery. Factors associated with recurrence and incontinence. Dis Colon Rectum. 1996;39(7):723−9.)
Sphincter-dividing procedures and extent of division	Procedure-related	Any strategy involving sphincter division can precipitate fecal incontinence; risk may rise with longer division length	Procedure class (fistulotomy/fistulectomy/cutting seton vs sphincter-sparing); quantified division length on EAUS or surgeon report	Fecal incontinence remains clinically relevant after sphincter division; severity increases with increasing length of division in imaging-based studies. (Bokhari S, Lindsey I. Incontinence following sphincter division for treatment of anal fistula. Colorectal Dis. 2010;12(7 Online):e135-9.)
Operative strategy (technique category)	Procedure-related	Technique choice may reflect both anatomy and planned sphincter preservation	Technique categories: fistulotomy, seton (cutting/draining), advancement flap, LIFT, plug, etc.	Postoperative fecal incontinence associated with “type of surgery” in large retrospective series; guideline documents emphasize technique selection to balance cure and function. (Garcia-Aguilar J, Belmonte C, Wong WD, et al. Anal fistula surgery. Factors associated with recurrence and incontinence. Dis Colon Rectum. 1996;39(7):723−9.)

**Abbreviations:** CCF-IS, Cleveland Clinic Florida Incontinence Score (Wexner); EAUS, endoanal ultrasonography; LIFT, ligation of intersphincteric fistula tract; MRI, magnetic resonance imaging; TRUS, transrectal ultrasonography. References correspond to the manuscript’s reference list in the full submission.

1. **Patient characteristics:** age; sex; body mass index (BMI) or obesity status; diabetes mellitus; smoking status; obstetric history (where available); preoperative continence status; history of prior anorectal surgery; history of prior fistula procedures; overall comorbidity burden.
2. **Fistula anatomy and complexity:** classification as “high” versus “low”; tract type (e.g., trans‑sphincteric, supra‑sphincteric); presence of complex features (horseshoe extensions, multiple tracts, secondary abscess cavities); location of the internal opening; anterior fistula in female patients.
3. **Operative factors:** surgical procedure (e.g., fistulotomy/fistulectomy, seton use [cutting or loose], advancement flap, LIFT, video‑assisted anal fistula treatment [VAAFT], laser closure [FiLaC], fistula plug/glue, combined or staged approaches); extent of sphincter division (if documented); surgery for recurrent fistula; occurrence of postoperative infectious complications.

### Comparator

For each prognostic factor, the comparator will be defined within each primary study as the absence of the factor (or a lower exposure category) versus its presence (or a higher exposure category).

### Outcomes

The definitions and measurement tools for the primary and secondary outcomes are outlined in **Table 2**.

**Table 2:** Outcome measures for postoperative fecal incontinence.

Outcome	Type	Definition	Measurement tool	Time point
Incidence of postoperative fecal incontinence	Primary	New-onset fecal incontinence or worsening fecal incontinence compared with preoperative baseline (as defined by each study; studies without baseline continence assessment analyzed separately where feasible)	Dichotomous (yes/no), clinician report or patient report; preferably anchored to validated scales	≥3 months post-op (minimum); primary analytical window 3–6 months; long-term analyses planned for ≥12 months
Fecal incontinence severity (symptom score)	Secondary	Severity level or change from baseline on validated scoring systems	Wexner/CCF-IS; Vaizey/St Mark’s	Study-specific follow-up; extracted at the 3- to 6-month window where possible and at ≥12 months for long-term follow-up
Continence-related quality of life	Secondary	Impact of fecal incontinence on daily activities and psychosocial domains	FIQL (Fecal Incontinence Quality of Life Scale)	Study-specific (preferably ≥3 months to reduce transient postoperative effects)

**Abbreviations:** CCF-IS, Cleveland Clinic Florida Incontinence Score; FIQL, Fecal Incontinence Quality of Life Scale; post-op, postoperative.

Primary outcome: Postoperative fecal incontinence, defined as new-onset fecal incontinence or a clinically meaningful worsening of continence relative to the preoperative baseline. Fecal incontinence must be assessed using a validated instrument (e.g., Wexner Score, Cleveland Clinic Florida Incontinence Score [CCF-IS], Vaizey/St. Mark’s Score) or clearly specified clinical criteria (e.g., involuntary loss of gas, liquid, or solid stool). To minimize misclassification from early postoperative seepage, studies must report fecal incontinence outcomes at least 3 months after surgery; where multiple time points are available, the 3- to 6-month assessment will be treated as the primary analytical window, with ≥12-month data examined as long-term follow-up. Studies lacking baseline continence assessment will not be excluded solely for this reason, but baseline assessment status will be extracted, reported, and examined through subgroup and sensitivity analyses.

Secondary outcomes include: severity of fecal incontinence (change in continuous scores from baseline); type-specific leakage (flatus, liquid, solid); daily pad use; urgency; and patient-reported quality-of-life measures specifically related to continence (if reported).

### Study design

Observational studies (prospective or retrospective cohort studies, case-control studies) that report quantitative associations between the candidate predictors and postoperative fecal incontinence will be included. Randomized controlled trials (RCTs) will generally be excluded, as their primary aim is to evaluate therapeutic efficacy rather than to assess prognostic associations in an unselected cohort. However, if an RCT presents a multivariable prognostic analysis across treatment arms (i.e., independent of randomization group) with extractable effect estimates, it may be considered in a sensitivity analysis.

### Information sources and search strategy

A comprehensive literature search will be performed in the following electronic databases from their inception to January 11, 2026: PubMed, Embase, Cochrane Library, and Web of Science. No restrictions on language or publication date will be applied. Supplementary searches will include grey literature repositories (e.g., OpenGrey, where accessible), relevant conference proceedings, and the reference lists of all included studies and pertinent systematic reviews.

The search strategy was developed in consultation with a medical information specialist and combines controlled vocabulary (e.g., Medical Subject Headings [MeSH] in PubMed, Emtree in Embase) with free-text terms. Before final execution, the PubMed strategy will be peer-reviewed by another experienced information specialist using the Peer Review of Electronic Search Strategies (PRESS) 2015 checklist [28]; any revisions arising from this peer review will be documented and reported in the final review. Key search concepts cover:

1. Anal fistula (e.g., “anal fistula”, “fistula‑in‑ano”, “perianal fistula”);
2. Surgery (e.g., “fistulotomy”, “seton”, “advancement flap”, “LIFT”, “VAAFT”, “laser”, “FiLaC”, “plug”, “glue”);
3. Fecal incontinence (e.g., “fecal incontinence”, “faecal incontinence”, “continence”, “Wexner”, “Vaizey”, “St Mark’s”);
4. Prognosis or risk (e.g., “risk factor”, “predictor”, “association”, “odds ratio”).

Boolean operators (AND, OR) will be used to combine these concepts. The full search strategy for PubMed is provided in Table 3, together with the planned PRESS peer-review process. Strategies for other databases will be adapted accordingly.

**Table 3:** Search strategy for PubMed (to be adapted for other databases).

Concept	MeSH terms	Free-text keywords (Title/Abstract)	Boolean operators
Anal fistula	“Anal Fistula”[MeSH]	anal fistula; fistula-in-ano; perianal fistula; cryptoglandular fistula	OR
Surgery	“Surgical Procedures, Operative”[MeSH]	surgery; operative; fistulotomy; fistulectomy; seton; cutting seton; draining seton; advancement flap; LIFT; plug; glue; VAAFT; laser; FiLaC	OR
Fecal incontinence	“Fecal Incontinence”[MeSH]	fecal incontinence; faecal incontinence; continence; incontinence; Wexner; Cleveland Clinic; Vaizey; St Mark*; FIQL	OR
Prognosis/risk factors	“Risk Factors”[MeSH] OR “Prognosis”[MeSH]	risk factor*; predictor*; prognostic factor*; associated; multivariable; odds ratio; hazard ratio	OR
Combined strategy	—	(#1 AND #2 AND #3 AND #4)	AND
Filters	—	Humans; adults (≥18 years); no date limit; no language restriction	—
Search peer review	—	PubMed strategy to be peer-reviewed using the PRESS 2015 checklist before final execution; revisions documented	**—**

**Abbreviations**: MeSH, Medical Subject Headings; PRESS, Peer Review of Electronic Search Strategies. The final strategy will be peer-reviewed using the PRESS 2015 checklist and translated for Embase, Web of Science, Cochrane Library, and grey literature sources.

### Study selection

All retrieved records will be imported into EndNote X9 (Clarivate Analytics) for removal of duplicates. The study selection will follow a two‑phase screening process conducted independently by two reviewers. First, titles and abstracts will be screened against the eligibility criteria. Second, the full texts of potentially relevant records will be assessed. Discrepancies at each stage will be resolved through discussion; if consensus cannot be reached, a third reviewer will arbitrate. The selection process will be documented using a PRISMA‑compliant flow diagram.

### Data extraction

Data will be extracted independently by two reviewers using a piloted, standardized electronic form. Any discrepancies will be resolved by consensus or third‑party adjudication. The following data items will be collected:

1. Study characteristics: first author, publication year, country, study design, recruitment period, sample size, and funding source.
2. Participant characteristics: mean or median age, sex distribution, baseline continence status (including whether continence was assessed preoperatively and which instrument was used), relevant comorbidities, history of prior anorectal or fistula surgery, and obstetric history (if reported).
3. Fistula characteristics: classification system used, complexity (e.g., high vs. low, trans‑sphincteric extent), number of tracts, presence of horseshoe extensions or abscesses, and location of the internal opening.
4. Surgical details: primary operative technique, use of setons (type and duration), whether the procedure was single‑stage or staged, and any documented extent of sphincter division.
5. Outcome assessment: definition of fecal incontinence, measurement instrument (e.g., Wexner score), timing of postoperative assessment (categorized as <3 months [early], 3–6 months [primary analytical window], and ≥12 months [long-term]), follow-up duration, baseline continence assessment status, and proportion of participants lost to follow-up.
6. Effect estimates: Adjusted effect estimates (odds ratio [OR], risk ratio [RR], or hazard ratio [HR] with 95% confidence intervals [CIs]) will be preferentially extracted, along with the covariates included in the model. All model covariates, including demographic, clinical, anatomical, and surgical variables, will be carefully recorded and reported to ensure transparency in data extraction and synthesis. Particular attention will be paid to whether models adjusted for fistula complexity, baseline continence, prior anorectal or fistula surgery, procedure type, and extent of sphincter division. If adjusted estimates are unavailable, unadjusted estimates will be recorded but synthesized separately and interpreted cautiously.

If key data are presented only in figures, WebPlotDigitizer (version 4.4) will be used to extract numerical values where feasible. Where necessary, corresponding authors will be contacted to obtain missing or clarificatory information.

### Risk of bias assessment

The risk of bias of included prognostic factor analyses will be assessed independently by two reviewers using the Quality in Prognosis Studies (QUIPS) tool [29], which is specifically designed for prognostic factor research and evaluates study participation, study attrition, prognostic factor measurement, outcome measurement, study confounding, and statistical analysis or reporting. For cohort or case-control studies in which QUIPS is not fully applicable, and for supplementary descriptive appraisal, the Newcastle-Ottawa Scale (NOS) will also be used as appropriate [30]. Domain-level judgments rather than only total scores will guide interpretation, particularly for confounding by fistula complexity, baseline continence, and operative technique. Any discrepancies between reviewers will be resolved through discussion or, if necessary, by adjudication from a third reviewer. Inter-rater agreement will be quantified using the Cohen’s kappa statistic.

### Certainty of evidence

The overall certainty of evidence for each identified prognostic factor will be evaluated using the Grading of Recommendations, Assessment, Development and Evaluation (GRADE) framework, adapted for prognostic factor studies [31]. We will assess the following domains: risk of bias, inconsistency, indirectness, imprecision, and publication bias. Evidence will initially be considered as high certainty for well-conducted observational studies and then downgraded based on shortcomings in any of the assessed domains. The final certainty for each factor will be categorized into one of four levels: high, moderate, low, or very low.

## Statistical analysis plan and data synthesis

### Effect measures and synthesis prioritization

Adjusted effect estimates (ORs, RRs, or HRs) will be prioritized for quantitative synthesis and pooled where appropriate, as they better account for confounding and are most relevant to prognostic inference. When multiple adjusted models are reported within a study, we will preferentially extract the estimate from the model judged to be most clinically and methodologically appropriate (typically the most fully adjusted model), and we will document the covariate set used. Where the research question involves operative technique, pooling will be restricted to estimates adjusted for key confounders wherever possible, especially fistula complexity, baseline continence status, prior anorectal or fistula surgery, age, sex, and extent of sphincter division. If procedure-specific estimates are not adjusted or stratified for fistula complexity, they will not be interpreted as causal effects of procedure type and may be summarized narratively or in separate sensitivity analyses.

Unadjusted effect estimates will not be pooled together with adjusted estimates in the primary meta-analyses. Where only unadjusted estimates are available, these will be synthesized separately (as a distinct analysis) and/or used in sensitivity analyses to explore the influence of confounding and the robustness of conclusions.

For dichotomous outcomes of fecal incontinence, measures of association as reported in the primary studies will be aggregated; conversion between different measures (e.g., RR to OR) will only be performed when baseline risk data are available and underlying assumptions are explicitly stated. Continuous outcomes, such as incontinence severity scores, will be pooled as mean differences when the same instrument is used across studies, or as standardized mean differences when different scales are employed. Change-from-baseline data will be utilized where reported.

### Meta-analysis approach and assessment of heterogeneity

Provided that studies are sufficiently homogeneous in clinical and methodological aspects, meta-analysis will be conducted using a generic inverse-variance random-effects model, which accounts for both within-study and between-study variability. Heterogeneity will be quantitatively assessed using the Cochran’s Q statistic (with a p-value <0.10 considered indicative of significant heterogeneity) and the I^2^ statistic. I^2^ values will be interpreted using conventional intervals: 0–30% (low), 30–50% (moderate), 50–70% (considerable), and 70–100% (substantial) [32]. Where statistical pooling is deemed inappropriate due to clinical or methodological diversity, or extreme heterogeneity, a structured narrative synthesis supported by summary tables will be presented.

### Planned subgroup analyses

If sufficient data are available, we will perform subgroup analyses to explore potential sources of heterogeneity. Planned subgroups include:

1. Fistula complexity (simple vs. complex; low vs. high trans‑sphincteric);
2. Surgical technique category (sphincter‑cutting vs. sphincter‑preserving procedures; single‑stage vs. staged operations);
3. Use of preoperative continence assessment (objective measurement vs. none);
4. Duration of follow-up (3–6 months, ≥ 12 months);
5. Definition and measurement of fecal incontinence (dichotomous outcome vs. validated score; variation in diagnostic thresholds).

### Sensitivity analyses

To examine the robustness of the pooled estimates, the following sensitivity analyses are planned:

- Restriction to studies judged to be at low risk of bias;
- Primary meta-analysis restricted to adjusted effect estimates only (i.e., excluding unadjusted estimates);
- Separate synthesis of unadjusted effect estimates (where available), and comparison with adjusted-effect syntheses to assess potential confounding;
- Exclusion of studies with mixed patient populations (including Crohn’s disease) where data specific to cryptoglandular fistulas cannot be extracted;
- Restriction to studies that employed validated instruments for assessing fecal incontinence;
- Restriction to studies with documented baseline continence assessment, with studies lacking baseline continence data analyzed separately where feasible;
- Restriction to studies reporting the primary fecal incontinence outcome at 3 months or later, excluding early-only assessments.

### Assessment of small-study effects and publication bias

For any meta-analysis comprising ten or more studies, potential small-study effects and publication bias will be evaluated. Funnel plots will be generated for visual inspection of asymmetry, supplemented by formal statistical testing using Egger’s regression test. Where asymmetry is suggested, the trim-and-fill method will be applied as an exploratory sensitivity analysis to estimate the potential effect of missing studies on the pooled estimate [33].

### Inter-rater agreement

Agreement between reviewers during study screening and risk‑of‑bias assessment will be quantified using Cohen’s kappa statistic, reported with corresponding 95% confidence intervals.

### Threshold for consistent association

To identify factors with robust evidence, a factor will be considered “consistently associated” with fecal incontinence only if the majority of contributing studies report effect estimates in the same direction and demonstrate statistical significance. The pre-specified ≥75% directional-consistency threshold is intended as a pragmatic descriptive criterion, not a formal statistical decision rule: it requires at least three quarters of contributing studies to point in the same direction, thereby reducing the risk that a single influential or isolated statistically significant study is over-emphasized. This criterion will be interpreted alongside pooled effect estimates, heterogeneity, risk of bias, adjustment for confounding, and certainty of evidence [34].

### Patient and public involvement

As this review synthesises existing published data, patients and the public were not directly involved in its design or conduct. The research question and outcome priorities, however, were informed by recognised clinical concerns regarding continence preservation and postoperative counselling encountered in routine practice.

### Ethics and dissemination

No ethical approval is required for this study, as it will analyse and synthesise previously published aggregate data. Findings will be disseminated through submission to a peer‑reviewed journal and presentations at relevant scientific conferences. To ensure transparency and reproducibility, supplementary materials—including full search strategies, data extraction forms, and evidence profile tables—will be made publicly available.

### Protocol amendments

Any modifications to this protocol will be documented and updated in the PROSPERO registration record. All amendments will be clearly described and justified in the final manuscript, together with an assessment of their potential impact on the review’s conclusions.

## Discussion

Postoperative fecal incontinence represents one of the most consequential functional trade-offs in the surgical management of anal fistula and is explicitly recognized as a critical patient-centered outcome in contemporary clinical guidelines [4,5]. While the choice of surgical technique, most notably fistulotomy, is a well-established risk factor, clinical experience and observational data suggest that postoperative fecal incontinence arises from a complex interplay of patient-specific vulnerability, fistula anatomy, and procedural details. This study are designed to synthesize this dispersed evidence, with the goal of moving from generalized awareness to a quantified, evidence-based understanding of prognostic factors.

### Expected findings and mechanistic context

We anticipate that the quantitative synthesis will most robustly support associations between postoperative fecal incontinence and factors indicative of diminished sphincteric reserve or greater anatomical complexity. These likely include female sex, pre-existing fecal incontinence or continence impairment, a history of prior fistula surgery (a “redo” setting), and high or complex fistula anatomy (e.g., high transsphincteric or suprasphincteric tracts, horseshoe extensions). Mechanistically, these factors are linked through several plausible pathways including direct structural compromise of the sphincter complex during tract eradication, cumulative injury from previous procedures leading to fibrosis and reduced compliance, and the unmasking of latent weakness in patients with subclinical pelvic floor dysfunction. Anatomy is a central moderator, as complex tracts often necessitate more extensive dissection or influence the selection of a sphincter-preserving technique, which itself may carry a variable risk profile.

### Positioning within the existing evidence landscape

Existing meta-analyses in this field have predominantly focused on comparative efficacy for healing and recurrence across procedures, with fecal incontinence often treated as a secondary safety endpoint or reported descriptively rather than synthesized as a primary outcome. In parallel, numerous cohort studies have examined predictors of postoperative fecal incontinence, but their findings remain fragmented across decades, with heterogeneous case-mix, outcome definitions, follow-up time points, and adjustment strategies that limit interpretability and preclude straightforward translation into counseling or decision support.

This study is positioned differently in both scope and outputs. First, fecal incontinence is explicitly the primary endpoint, and the unit of synthesis is the prognostic factor (patient-, disease-, and procedure-related) rather than the procedure comparison. Second, beyond pooling effect estimates where clinically comparable, we will produce factor-specific judgments about the credibility and certainty of evidence (e.g., using a prognostic-question-appropriate certainty framework), allowing clinicians to distinguish factors supported by consistent, adjusted evidence from those driven by sparse or high-bias data. Third, the synthesis is explicitly risk-stratification oriented: by mapping which factors (and combinations of factors, where data permit) are most informative for predicting new-onset or worsening fecal incontinence, the study will aim to move the field from broad awareness (e.g., complex fistulas are riskier) toward more clinically actionable, quantified prognostic statements that can guide individualized counseling and procedure selection. In doing so, the study will complement, rather than duplicate, prior technique-focused syntheses, by providing an evidence-graded prognostic framework that can be used to identify high-risk subgroups, prioritize baseline continence assessment and imaging, and highlight where additional primary research is most needed.

### Addressing methodological challenges

We anticipate several sources of heterogeneity that will require careful handling. These include variability in the definition and measurement of fecal incontinence (e.g., binary outcome vs. validated score, differing thresholds) and the timing of postoperative assessment. We also anticipate confounding by indication and selection bias, whereby the choice of surgical technique is not random but is closely linked to fistula complexity and baseline patient vulnerability (e.g., pre-existing continence impairment, sphincter integrity, prior anorectal surgery, comorbidities, or clinician preference and local expertise). In this context, sphincter-preserving procedures may be preferentially selected for patients perceived to be at higher risk of postoperative fecal incontinence, while sphincter-cutting approaches may be reserved for less complex disease-potentially biasing comparisons in either direction.

Our protocol is designed to mitigate these issues through pre-specified strategies. We will prioritize adjusted effect estimates from primary studies and document the covariate sets to improve transparency regarding confounding control. We will conduct subgroup analyses based on fistula complexity, surgical technique category, baseline continence assessment, and outcome measurement tools, and interpret pooled estimates cautiously where residual confounding is likely. We will not treat procedure type as independent of fistula complexity unless the primary study has adjusted for or stratified by complexity and other key covariates. Where statistical pooling is not feasible due to clinical or methodological diversity, or where bias structures differ materially across studies, we will employ a structured narrative synthesis supported by summary tables. Sensitivity analyses, including restriction to studies at low risk of bias, studies with baseline continence assessment, and analyses limited to adjusted estimates, will further test the robustness of findings to confounding by indication and selection processes, as well as to variations in study quality and outcome ascertainment.

### Strengths and limitations

The principal strengths of this study lie in its comprehensive, multi-database search strategy, dual-independent review processes, adherence to PRISMA-P standards, and a pre-specified analytic plan that extends beyond procedural comparisons to integrate patient- and fistula-level factors [26]. However, important limitations are inherent to the evidence base. The predominance of observational studies implies that residual confounding is an inherent constraint and cannot be fully eliminated, even when adjusted estimates are pooled, particularly where procedure choice is driven by fistula complexity and baseline continence vulnerability. As a result, any observed associations will be interpreted as prognostic or associational rather than causal, and we will emphasize the potential direction and plausibility of residual confounding when drawing conclusions. Heterogeneity in outcome reporting and potential publication bias, where null findings for fecal incontinence may be under-reported, may influence the summary estimates. The deliberate exclusion of fistulas related to Crohn’s disease ensures focus on cryptoglandular etiology but also limits the generalizability of findings to inflammatory bowel disease populations. In grading the certainty of the evidence, we anticipate rating down for risk of bias and confounding where appropriate, and we will reflect residual confounding and selection processes in the GRADE certainty ratings (e.g., downgrading for study limitations and, where relevant, inconsistency and imprecision). Consequently, overall certainty is expected to be low to very low for many comparisons, and recommendations will be framed cautiously in line with this level of certainty.

### Clinical implications and future directions

Identifying consistent, graded risk factors has direct clinical utility. If factors such as baseline continence impairment, high or complex fistula anatomy, prior fistula surgery, female sex, older age, or extensive sphincter division are confirmed as consistent predictors, the review may support a practical preoperative pathway: routine baseline continence scoring, selective sphincter imaging, explicit patient counseling on long-term fecal incontinence risk, and preference for sphincter-preserving or staged strategies in high-risk patients. Beyond immediate practice, our findings are expected to highlight key gaps in the literature. Future primary studies would be strengthened by the routine use of standardized, patient-reported continence instruments (e.g., Wexner score), consistent application of fistula classification systems based on preoperative imaging, explicit reporting of the extent of sphincter division, and longer-term follow-up to distinguish transient postoperative symptoms from persistent dysfunction. Ultimately, this work aligns with initiatives to develop core outcome sets for anal fistula surgery, ensuring that functional outcomes such as fecal incontinence are measured and reported with the same rigor as traditional metrics of healing [35].

## Conclusion

This protocol details the planned conduct of a systematic review and meta-analysis that will synthesize evidence on patient-, disease-, and surgery-related factors associated with fecal incontinence following surgery for cryptoglandular anal fistula. By integrating quantitative evidence synthesis with a formal assessment of certainty, this review aims to translate the currently fragmented understanding of continence risk into an evidence-weighted foundation. The findings are anticipated to support more personalized risk stratification, improve patient-centered counseling, and inform procedure selection for patients at increased risk of postoperative fecal incontinence, ultimately contributing to improved functional outcomes in this challenging clinical context.

## Supporting information

S1 Table PRISMA-P checklist. (DOC)

## Abbreviations

BMI: Body Mass Index
CCF-IS: Cleveland Clinic Florida Incontinence Score
CI: Confidence Interval
EAUS: Endoanal Ultrasonography
FI: Fecal Incontinence
FIQL: Fecal Incontinence Quality of Life Scale
FiLaC: Fistula-tract Laser Closure
GRADE: Grading of Recommendations Assessment, Development and Evaluation
HR: Hazard Ratio
LIFT: Ligation of the Intersphincteric Fistula Tract
MRI: Magnetic Resonance Imaging
NOS: Newcastle-Ottawa Scale
OR: Odds Ratio
PICOS: Population, Intervention/Index, Comparator, Outcome, Study design
PRISMA-P: Preferred Reporting Items for Systematic Review and Meta-Analysis Protocol
PRESS: Peer Review of Electronic Search Strategies
QUIPS: Quality in Prognosis Studies
RR: Relative Risk
TRUS: Transrectal Ultrasonography
VAAFT: Video-Assisted Anal Fistula Treatment
